# Ecological stoichiometry influences phytoplankton alpha and beta diversity rather than the community stability in subtropical bay

**DOI:** 10.1002/ece3.9301

**Published:** 2022-09-09

**Authors:** Qiangsheng Xu, Meiqin Huang, Shu Yang, Xiaoli Li, Huaxian Zhao, Jinli Tang, Gonglingxia Jiang, Zhuoting Li, Yuqing Huang, Ke Dong, Liangliang Huang, Nan Li

**Affiliations:** ^1^ Key Laboratory of Ministry of Education for Environment Change and Resources Use in Beibu Gulf (Nanning Normal University), Guangxi Key Laboratory of Earth Surface Processes and Intelligent Simulation Nanning Normal University Nanning Guangxi China; ^2^ Guangxi Station of Radiation Environment Supervision, Department of Ecology and Environment of Guangxi Nanning China; ^3^ School of Agriculture, Ludong University Yantai China; ^4^ Department of Biological Sciences Kyonggi University Suwon‐si South Korea; ^5^ College of Environmental Science and Engineering, Guilin University of Technology Guilin China

**Keywords:** Beibu gulf, community stability, distribution, ecological stoichiometry, phytoplankton diversity, *rbcL* gene

## Abstract

Numerous studies have shown that changes in environmental factors can significantly impact and shift the structure of phytoplankton communities in marine ecosystems. However, little is known about the association between the ecological stoichiometry of seawater nutrients and phytoplankton community diversity and stability in subtropical bays. Therefore, we investigated the relationship between the phytoplankton community assemblage and seasonal variation in the Beibu Gulf, South China Sea. In this study, we found that the abundance of Bacillariophyceae in spring was relatively greater than in other seasons, whereas the abundance of Coscinodiscophyceae was relatively low in spring and winter but greatly increased in summer and autumn. Values of the alpha diversity indices gradually increased from spring to winter, revealing that seasonal variations shifted the phytoplankton community structure. The regression lines between the average variation degree and the Shannon index and Bray–Curtis dissimilarity values showed significantly positive correlations, indicating that high diversity was beneficial to maintaining community stability. In addition, the ecological stoichiometry of nutrients exhibited significantly positive associations with Shannon index and Bray–Curtis dissimilarity, demonstrating that ecological stoichiometry can significantly influence the alpha and beta diversity of phytoplankton communities. The C:N:P ratio was not statistically significantly correlated with average variation degree, suggesting that ecological stoichiometry rarely impacted the community stability. Temperature, nitrate, dissolved inorganic phosphorous, and total dissolved phosphorus were the main drivers of the phytoplankton community assemblage. The results of this study provide new perspectives about what influences phytoplankton community structure and the association between ecological stoichiometry, community diversity, and stability in response to environmental changes.

## Graphical abstract

Seasonal change significantly shifted the phytoplankton community structure, and high diverse community was beneficial to maintaining the community stability. ;Ecological stoichiometry significantly affected the community diversity, and temperature, NO3‐, DIP and TDP were the main drivers of the community assemblage. ;This study provide new perspectives about what influences phytoplankton community structure and the association between ecological stoichiometry, community diversity, and stability in response to environmental changes in subtropical bay.

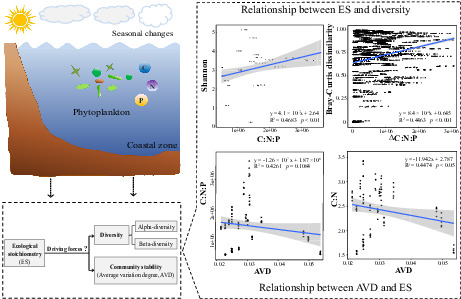

## 1 INTRODUCTION

Phytoplankton are the most important primary producers in marine ecosystems and play a crucial role in biogeochemical cycling and food webs (Field et al., 1998; Li et al., 2021). Phytoplankton are the first link in the trophic chain and perform the significant function of organic material transfer between all abiotic and biotic components of the oceanographic environment (Staniszewska et al., 2015). The highly diverse nature of marine phytoplankton helps maintain the ocean's ecological balance because these species contribute to stability of material and energy cycling in marine ecosystems (Kosek et al., 2016).

Phytoplankton freely live in marine seawater, and thus their community structure and diversity are easily susceptible to environmental perturbations (Kosek et al., 2016; Li et al., 2021; Liang et al., 2020). Temperature acts as an important environmental factor to affect the phytoplankton community structure. For example, it could regulate Antarctic phytoplankton community composition and size structure (Biggs et al., 2019). The general trend of phytoplankton succession and the community were interrupted due to the effects of elevated temperatures of thermal discharge from a power plant on coastal waters of the Bohai Sea off Qinhuangdao, China (Dong et al., 2021). Nutrient factors also play a crucial role in affecting the phytoplankton community. Nutrient variables were found to significantly influence the phytoplankton community structure in every season in the estuary around Luoyuan Bay, China (Pan et al., 2017). Microcosm experiments conducted in Sanya Bay in the northern South China Sea revealed that enhancement of dissolved organic carbon (DOC) content could lead to a shift in the phytoplankton community and composition (Liao et al., 2019). Seasonal variability of nitrogen content influenced and regulated phytoplankton community structure in the eastern Arabian Sea (Shetye et al., 2019). Therefore, water temperature and nutrient content jointly influenced the marine phytoplankton community structure (Fu et al., 2016; Patoucheas et al., 2021; Zhang et al., 2016).

These shifts in phytoplankton composition and structure in response to environmental disturbance result in two main types of deterministic and stochastic processes in aquatic ecosystems (Bandyopadhyay et al., 2008; Jang & Allen, 2015; Mandal & Banerjee, 2013; Xue et al., 2018). The community stability has been attributed principally to species diversity because the general consensus is that biodiversity has positive effects on the community stability (Loreau & de Mazancourt, 2013; Vallina et al., 2017; Xun et al., 2021). The community stability can be evaluated using average variation degree (AVD), which is calculated as the degree of deviation from the average value of the relative abundance of normally distributed operational taxonomic units (OTUs) in variable environmental conditions, and a low AVD value represents high community stability (Xun et al., 2021). Similarly, phytoplankton community stability might be primarily determined by phytoplankton species diversity, which requires evaluation of the relationship between phytoplankton diversity and stability in marine ecosystems. Meanwhile, assessment of ecological stoichiometry (ES) mainly focuses on chemical elements (carbon (C), nitrogen (N), and phosphorous (P)) in components as well as interactions and processes in ecosystems, and it is beneficial for understanding the effect of human activities on the balance and biogeochemical cycling of bio‐elements in oceanographic ecosystems (Babbin et al., 2014; Bradshaw et al., 2012; Chen et al., 2021). Consequently, the number of ES studies has increased rapidly in recent years (Sardans et al., 2021). For example, Hillebrand et al. (2013) found that N:P ratios of phytoplankton decreased as their growth rate increased and variance decreased, which means that fast‐growing phytoplankton contained more P and had a simpler elemental composition (Hillebrand et al., 2013; Sardans et al., 2021). However, little is known about the association between the ecological stoichiometry of seawater nutrients and phytoplankton community diversity and stability in subtropical bays.

To better understand the key factors that regulate shifts in phytoplankton structure and community stability at spatio‐temporal scales, this study analyzed seawater samples from the subtropical coastal waters of Beibu Gulf during four seasons using high‐throughput sequencing technology in order to (a) evaluate changes in the community structure of marine phytoplankton among seasons, (b) elucidate the potential relationships between phytoplankton community stability and various environmental factors, and (c) uncover the key factors that impact phytoplankton community stability in this subtropical bay. Our hypothesis was that the ecological stoichiometry of seawater nutrients might influence phytoplankton community diversity and stability in the subtropical bays.

## 2 MATERIALS AND METHODS

### 2.1 Study area and sample collection

The study was carried out in Maowei Sea, which is located in the coast of Beibu Gulf, northwest of South China Sea with subtropical climate. Figure S1 shows the specific locations of the sampling sites. GPS co‐ordinates of five sampling sites are M1 (108°32′38″ E, 21°50′8″ N), M2 (108°32′33″ E, 21°49′20″ N), M3 (108°32′30″ E, 21°48′7″ N), M4 (108°33′33″ E, 21°44′34″ N), and M5 (108°34′27″ E, 21°44′11″ N). Surface seawater samples used in this study were collected from a water depth of 0.5 m in the Maowei Sea using a rosette of Niskin bottles on 10 July, 2017 (summer), 9 September, 2017 (autumn), 11 December, 2017 (winter), and 10 March, 2018 (spring). Five seawater samples were collected at each of the five sampling sites. In total, 100 surface seawater samples were collected in the Maowei Sea during the study. Water temperature, pH, and salinity of each sample were measured using a portable meter (556 MPS; YSI, Yellow Springs, OH, USA). Following collection, samples were stored on ice for transport to the laboratory. For DNA extraction, 5 L of surface seawater were filtered sequentially through a 200 μm nuclepore polycarbonate filter to remove debris and larger organisms followed by a 0.22‐μm Millipore filter. The 0.22‐μm filters were stored at −20°C for subsequent analysis.

### 2.2 Environmental factors and nutrient analysis

Concentrations of nitrate (${\mathrm{NO}}_{3}^{-}$), nitrite (${\mathrm{NO}}_{2}^{-}$), ammonium (${\mathrm{NH}}_{4}^{+}$), and phosphate (PO_4_^3−^) were measured using spectrophotometric and colorimetric methods (Han et al., 2012). The chlorophyll *a* (Chl‐*a*) concentration was measured using spectrophotometry (American Public Health Association (APHA), 1999). Total organic carbon (TOC) content was measured using a TOC analyzer (TOC‐VCPH). Chemical oxygen demand (COD) was detected using the alkaline KMnO_4_ method. Dissolved oxygen (DO) was measured by the Winkler method using a digital DO meter (HQ30d, HACH, USA) (Shriwastav et al., 2017). Total dissolved nitrogen (TDN) and total dissolved phosphorus (TDP) contents were determined using a Lachat Quickchem 8500 flow injection analyzer (HACH). Dissolved inorganic nitrogen (DIN) content was calculated by summing the concentrations of ${\mathrm{NO}}_{2}^{-}$, ${\mathrm{NO}}_{3}^{-}$, and ${\mathrm{NH}}_{4}^{+}$. Dissolved inorganic phosphorus (DIP) level was estimated using the concentration of PO_4_^3−^‐P (Lai et al., 2014; Li et al., 2020). Seawater ES, including the ratios of C:N, C:P, N:P, and C:N:P, was calculated as molar ratios based on the TOC, TDN, and TDP. The results for the measured environmental parameters are shown in Table S1.

### 2.3 DNA extraction, PCR amplification, and high‐throughput sequencing

Total DNA was extracted from the filters using a DNeasy Power Water Kit (Qiagen) according to the manufacturer's procedures. DNA yield and purity were measured using a NanoDrop Spectrophotometer (Thermo Fisher Scientific). Phytoplankton *rbcL* gene fragments (550 base pairs [bp]) were amplified with previously published *rbcL* primers (F:5′‐GATGATGARAAYATTAACTC‐3′; R:5′‐ATTTGDCCACAGTGDATACCA‐3′) (John et al., 2007; Xu et al., 2015). For PCR, 2 μl of template DNA were aliquoted into illustraTM PuReTaq Ready‐To‐GoTM PCR Beads (GE Healthcare, Waukesha, WI, USA) with 21.5 μl of PCR‐grade molecular water and 1.5 μl of primer for a total reaction volume of 25 μl. PCR was conducted on a Bio‐Rad thermocycler (Hercules, CA, USA) under the following conditions: 1 min initial denaturation at 95°C, 35 cycles of 30 s denaturation at 95°C, 30 s annealing at 52°C, 1 min elongation at 72°C, and a final extension for 10 min at 72°C. Throughout the DNA extraction process, ultrapure water, instead of a sample solution, served as a negative control to exclude the possibility of false‐positive PCR results. Three technical replicates of the PCR reaction were conducted for each sample and prepared for MiSeq sequencing. The PCR products were purified using AMPure XT beads (Beckman Coulter Genomics). The reaction products were confirmed with 2% agarose gel electrophoresis and with a Nanodrop 2000 Spectrophotometer (Thermo Fisher Scientific). The PCR products for sequencing were prepared using a TruSeq DNA kit (Illumina) according to the manufacturer's instructions. The products from all samples were mixed at equal molar amounts and sequenced using an Illumina Miseq sequencer at Lianchuan‐Bio‐Technology Co., Ltd.

Raw sequences were processed and verified using the software package QIIME2 (Quality Insights Into Microbial Ecology) to remove sequences with primer mismatches or length < 275 bp, low‐quality reads (quality scores <30), primers, and barcode sequences (Caporaso et al., 2010; Rai et al., 2019). Chimeric sequences were identified and eliminated using UCHIME (Edgar et al., 2011). The software was further subjected to OTU clustering based on 97% sequence similarity. The representative sequences were annotated using a local blastn program and the ribosomal database project database (Release 11) (Cole et al., 2014). Taxonomic assignments of phytoplankton were performed using an available *rbcL* sequence database generated from GenBank data. Sequencing data were obtained from all 100 samples, and a total of 1,687,350 sequences, with a mean of 16,874 ± 21,756 in each sample, were retained after removing low‐quality reads (Table S2). The mean number of OTUs per sample was 298 ± 242 (Table S2). The coverage of sequencing samples was mostly >98% (Table S2). All phytoplankton sequencing data in FASTQ format were deposited in GenBank under access numbers ranging from SAMN20371288 to SAMN20371387 and Bioproject number PRJNA749375.

### 2.4 Data and statistical analyses

To illustrate the scope of phytoplankton diversity, Good's coverage (C) was calculated as [1 – (n/N)], where n is the number of OTUs that was observed once and N is the total number of OTUs in the sample. The statistical analyses in this study were mainly performed in R with the vegan, picante, pheatmap, and psych' packages. Alpha and beta diversity, analysis of similarities (ANOSIM), and permutational multivariate analysis of variance (PERMANOVA) analyses were conducted using the vegan package, and the psych package was used for data correlation analysis. The difference analyses were conducted using one‐way ANOVA. Correlation analyses were performed using Spearman's rank method. Alpha diversity was estimated using the Shannon, Simpson, Chao1, and observed number indices. Community comparison of phytoplankton assemblages (beta diversity) was conducted using Bray–Curtis distance and principal coordinate analysis (PCoA). Phytoplankton community stability was evaluated by AVD, which was calculated using the degree of deviation from the mean of the relative abundance of normally distributed OTUs among different seasons (Xun et al., 2021). Significant differences were defined as *p* < .05 or *p* < .01.

## 3 RESULTS

### 3.1 Abundance and spatial distribution of phytoplankton communities in Beibu gulf during four seasons

The abundance of phytoplankton taxa in the communities was analyzed at the class level (Figure S2). For all samples and all four seasons, Bacillariophyceae, Coscinodiscophyceae, Mediophyceae, Bangiophyceae, and Fragilariophyceae were the main abundant species and primarily constituted the phytoplankton community structure in the Beibu Gulf. However, the phytoplankton composition showed characteristic changes in structure during the different seasons. The abundance of Bacillariophyceae in spring was relatively greater than in other seasons, in which only some sampled sites displayed high abundance. The abundance of Coscinodiscophyceae in spring and winter was relatively low but greatly increased in summer and autumn, with mean percentages of 44.9 ± 25.6% and 36.8 ± 35.3%, respectively. The mean abundance of Fragilariophyceae during the spring was relatively greater than in other seasons. The abundance of Bangiophyceae was significantly higher in the winter, with a mean of 10.2 ± 11.9%, compared with the other seasons. In addition, the mean abundance of Mediophyceae in autumn and winter was relatively greater than in spring and summer. Interestingly, a relatively high abundance of Eustigmatophyceae (19.5%) was observed station SP1 in the spring (Figure S2).

**FIGURE 1:**
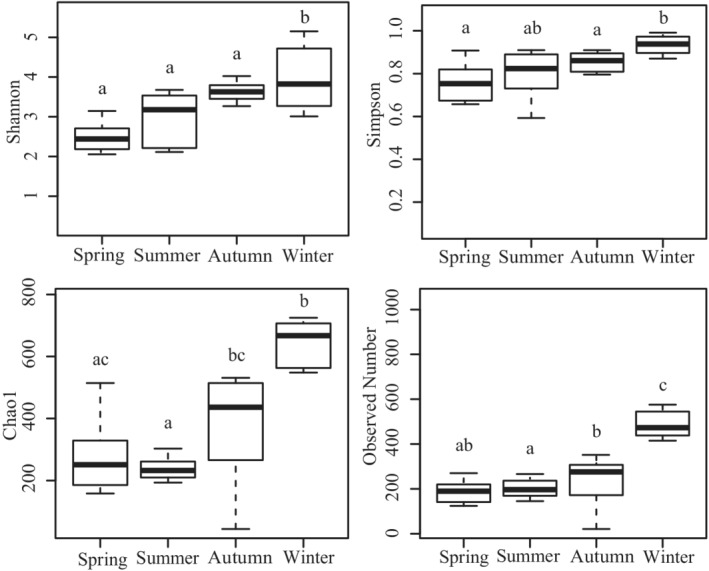
Alpha diversity indices (Shannon, Simpson, Chao1, and observed number) for samples collected in spring, summer, autumn, and winter. The values represent the mean of five samples for each group.

When analyzing alpha diversity, the Shannon diversity index of phytoplankton varied greatly from 5.15 to 0.23, with a mean value of 3.07 ± 1.11, among all of the samples (Table S2). Values of the Simpson diversity index varied from 0.98 to 0.07, with a mean value of 0.78 ± 0.22, among all samples (Table S2). The mean value and range of the Chao 1 index were 393.14 ± 282.32 and 1126.63 to 44.00, respectively (Table S2). The Shannon and Chao 1 index values were relatively high, demonstrating that phytoplankton communities in different sampling areas were quite abundant. Interestingly, the alpha diversity values for the Shannon, Simpson, and Chao 1 indices were highest in winter (Table S2). Moreover, all four alpha diversity indices had the highest median value in winter, which gradually decreased from winter to spring (Figure 1).

**FIGURE 2:**
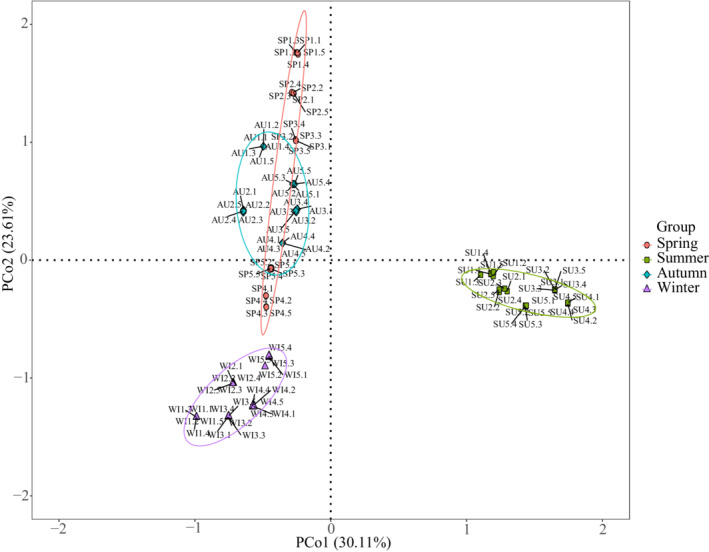
PCoA results showing the phytoplankton community variations based on Bray–Curtis distance matrices. Samples from spring, summer, autumn, and winter are labeled with red dots, green squares, blue rhombi, and purple triangles, respectively

The first two principal coordinates, PCo1 and PCo2, explained 30.11% and 23.61% of the total variance, respectively (Figure 2). The ANOSIM (*R* = 0.595, *p* = .001) and PERMANOVA (*R* ^2^ = 0.289, *p* = .001) tests also showed significant differences in phytoplankton community structure among the four seasons.

### 3.2 Relationship between AVD and Shannon index and Bray–Curtis dissimilarity in Beibu gulf during seasonal shifts

**FIGURE 3:**
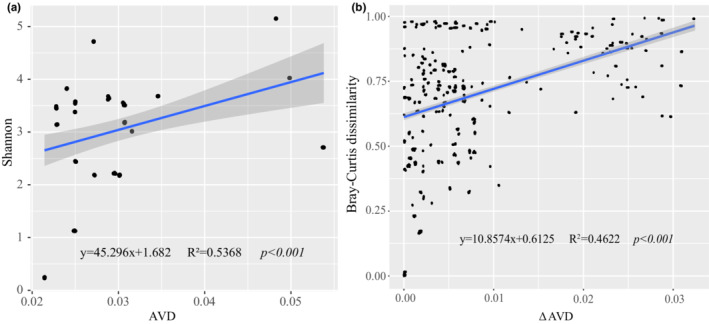
Results of linear regression analysis of AVD vs. Shannon index values (a) and of delta AVD vs. Bray–Curtis dissimilarity values (b). Straight lines represent linear relationships, and *p*‐values indicate significant differences

The relationships between AVD and the Shannon index and Bray–Curtis dissimilarity are shown as Figure 3. AVD was strongly positively correlated with Shannon index values (*p* < .001), and delta AVD was positively correlated with Bray–Curtis dissimilarity values (*p* < .001). These results show that AVD might be closely related to these metrics for the phytoplankton community.

### 3.3 Ecological stoichiometry effects on the phytoplankton community

**FIGURE 4:**
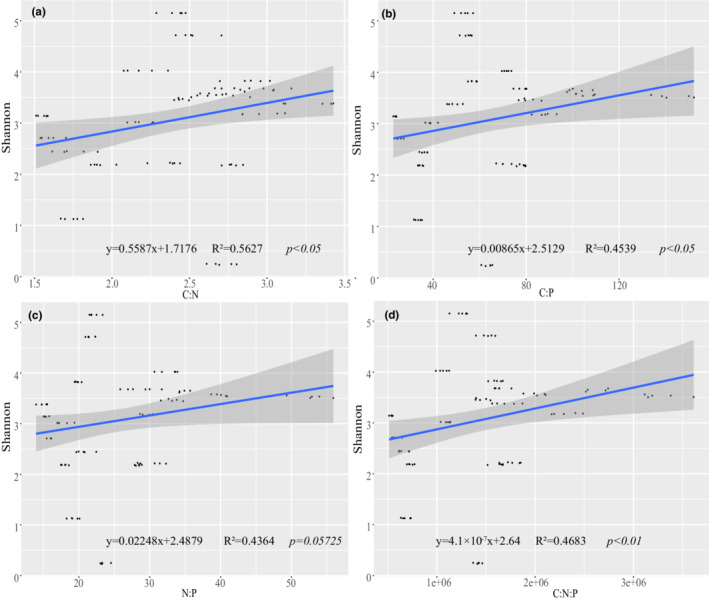
Results of linear regression analysis between the Shannon index values and nutrient ES of seawater. (a) Shannon index vs. C:N; (b) Shannon index vs. C:P; (c) Shannon index vs. N:P; (d) Shannon index vs. C:N:P. straight lines represent linear relationships, and *p*‐values indicate significant differences

**FIGURE 5:**
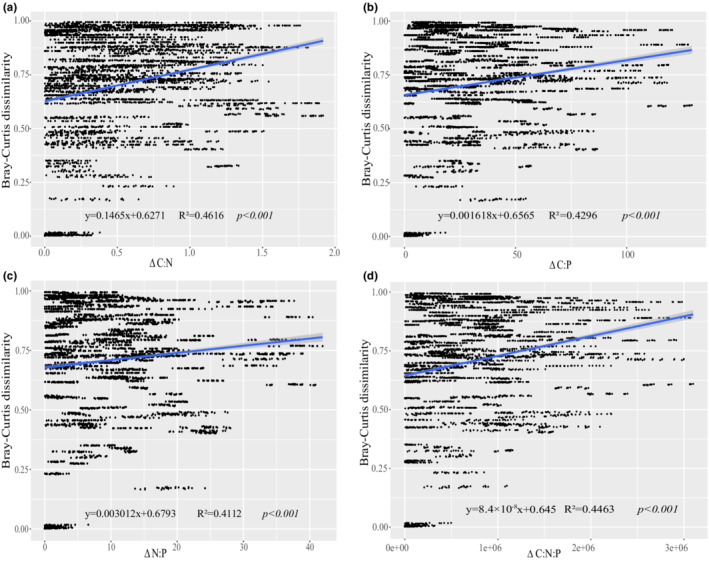
Results of linear regression analysis between Bray–Curtis dissimilarity values and nutrient ES of seawater. (a) Bray–Curtis dissimilarity vs. the delta ratio of C:N; (b) Bray–Curtis dissimilarity vs. the delta ratio of C:P; (c) Bray–Curtis dissimilarity vs. the delta ratio of N:P; (d) Bray–Curtis dissimilarity vs. the delta ratio of C:N:P. straight lines represent linear relationships, and *p*‐values indicate significant differences.

The mean values of ES ratios (C:N, C:P, N:P, and C:N:P) were highest in summer, and they gradually decreased from summer to spring to their lowest values (Table S1). The Shannon index values were significantly positively correlated with all four ratios as follows: C:N (*p* < .05), C:P (*p* < .05), N:P and C:N:P (*p* < .01) (Figure 4). All four ratios also were significantly positively correlated with Bray–Curtis dissimilarity values (*p* < .001) (Figure 5). In contrast, AVD was negatively correlated with all four ratios, but the correlation was only significantly negative for C:N (*p* < .05) (Figure S3).

### 3.4 Environmental factors that explain spatial variability in phytoplankton communities

The Mantel test and partial Mantel test revealed that environmental and biogeochemical factors and ratios of ES were significantly correlated with beta diversity of phytoplankton communities (Table 1). Salinity and TDP were significantly positively correlated with phytoplankton community beta diversity at the whole combined sample level and at the individual seasonal sampling level (Table 1). Generally, TDP, ${\mathrm{NO}}_{3}^{-}$, DIP, and temperature were the main factors that drove the phytoplankton community diversity (Table 1).

**TABLE 1:** Mantel and partial test analysis of environmental and biochemical factors

Environmental factors	Whole		Spring		Summer		Autumn		Winter	
	Mantel	Partial mantel	Mantel	Partial mantel	Mantel	Partial mantel	Mantel	Partial mantel	Mantel	Partial mantel
Temp	0.326***	0.206***	−0.054	−0.033	0.168*	0.045	−0.065	−0.056	−0.003	−0.014
pH	0.057***	0.03*	−0.048	0.000	−0.025	−0.010	−0.043	−0.064	0.116*	0.195**
Salinity	0.234***	0.068***	0.556***	0.321***	0.788***	0.556***	0.626***	0.461***	0.471***	0.293***
DO	0.299***	0.138***	0.079	−0.009	0.203**	0.060	0.352***	0.288***	0.247***	0.146**
NO_2_^−^	0.234***	0.061***	0.658***	0.341***	0.677***	−0.196	0.289***	0.095	0.473***	−0.118
NO_3_^−^	0.349***	0.246***	0.586***	0.495***	0.768***	0.361***	0.559***	0.202**	0.441***	−0.044
${\mathrm{NH}}_{4}^{+}$	0.272***	0.094***	0.593***	0.168**	0.713***	−0.033	0.471***	0.008	0.386***	0.159**
Chl‐*a*	0.227***	0.058**	0.448***	0.349***	0.837***	0.551***	0.533***	0.031	0.376***	0.376***
TDN	0.257***	−0.032	0.413***	0.402***	0.625***	0.29***	0.565***	0.238***	0.413***	0.269***
DIN	0.348***	0.165***	0.656***	0.43***	0.674***	0.357***	0.561***	−0.150	0.496***	0.047
DIP	0.361***	0.221***	0.531***	0.063	0.221*	0.099	0.355***	0.423***	0.657***	0.396***
TDP	0.321***	0.342***	0.307***	0.143**	0.744***	0.677***	0.447***	0.333***	0.396***	0.425***
TOC	0.29***	0.083***	0.62***	0.288***	0.677***	0.253***	0.493***	0.126*	0.544***	0.333***
COD	0.263***	0.064***	0.599***	0.194***	0.711***	0.099	0.566***	0.144*	0.54***	0.114*
C:N	0.213***	0.159***	0.020	−0.066	0.148*	−0.054	0.416***	0.181**	0.34***	0.093*
C:P	0.198***	0.010	0.612***	0.089*	0.455***	−0.002	0.455***	−0.301	0.54***	0.117*
N:P	0.244***	0.033*	0.647***	0.149**	0.462***	0.030	0.398***	−0.305	0.584***	0.193**
C:N:P	0.159***	−0.010	0.605***	0.078*	0.568***	−0.199	0.549***	0.018	0.536***	0.135*

*Note*: *p < .05; **p < .01; ***p < .001.

**FIGURE 6:**
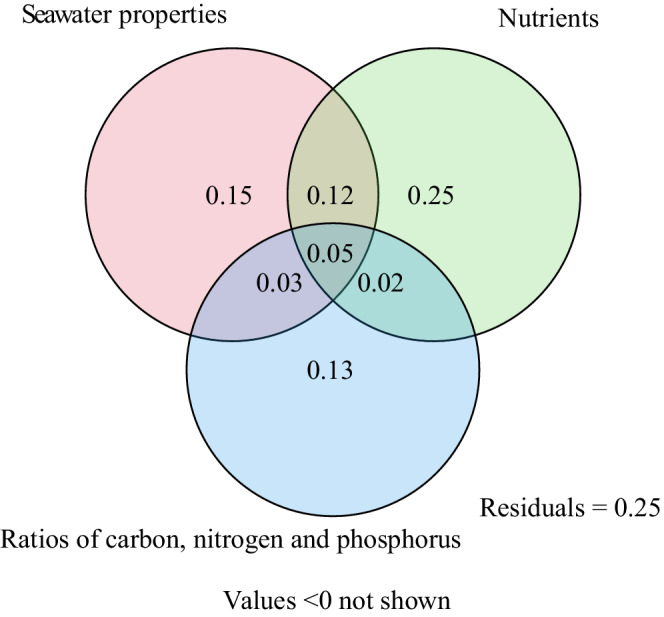
Results of VPA of the influences of seawater properties (temperature, pH, salinity, COD, and Chl‐*a* and dissolved oxygen contents), nutrient contents (${\mathrm{NO}}_{2}^{-}$, ${\mathrm{NO}}_{3}^{-}$, ${\mathrm{NH}}_{4}^{+}$, DIN, TDN, DIP, TDP, and TOC), and seawater nutrient ES (C:N, C:P, N:P, and C:N:P) in seawater from the Beibu gulf on phytoplankton community composition.

Seawater properties (temperature, pH, salinity, Chl‐*a*, dissolved oxygen, and COD), nutrients (${\mathrm{NO}}_{2}^{-}$, ${\mathrm{NO}}_{3}^{-}$, ${\mathrm{NH}}_{4}^{+}$, DIN, TDN, DIP, TDP, and TOC), and ratios of C:N, C:P, N:P, and C:N:P were able to explain approximately 75% of phytoplankton community variations (Figure 6). In addition, seawater properties, nutrient variables, and ratio values could independently account for 15%, 25%, and 13% of the total variation, respectively. Additionally, mutual interactions between seawater properties and nutrient variables were responsible for 12% of the variation, which was much higher than those between nutrients and ES ratios (2%) and seawater properties and ES ratios (3%).

## 4 DISCUSSION

In this study, we investigated variations in phytoplankton community structure in the subtropical Beibu Gulf area. We assessed phytoplankton community stability in response to environmental changes and identified the main drivers of the observed changes community structure. Bacillariophyceae, Coscinodiscophyceae, Mediophyceae, Fragilariophyceae, and Bangiophyceae were the dominant phytoplankton classes. Diatoms represent the richest group of autotrophic phytoplankton present in fresh, brackish, and marine waters worldwide, and they may be responsible for 20% of global photosynthetic carbon fixation in marine ecosystems (Mann et al., 2017). In our study, the greatest mean abundance of Bacillariophyceae occurred in spring and winter, which might be due to the relatively lower water temperature in these seasons in the Beibu Gulf area. Gogoi et al. (2021) also reported a negative correlation between Bacillariophyceae and water temperature in Sundarban waters. Interestingly, we found that mean abundance of Coscinodiscophyceae was higher in summer and autumn when the seawater temperature was elevated compared with other seasons. Similarly, in their study of the variations of phytoplankton community diversity in four different seasons in the coastal East China Sea, Xu et al. (2015) found that Coscinodiscophyceae diatoms dominated the summer samples. Thus, relatively high water temperature might be most suitable for the growth of Coscinodiscophyceae. Phytoplankton community structure and diversity were governed by dominant species as long as these species were not influenced by environmental disturbances. Compensatory growth of rare species occurred when environmental perturbations inhibited the growth of dominant species (Flöder et al., 2010). Abundances of these dominant species in our study varied in different seasons due to the environmental changes, which impacted the phytoplankton structure and community stability of the ecosystem (Flöder et al., 2010).

Values of all four of the alpha diversity indices gradually increased from spring to winter and exhibited the highest values in winter. Similarly, the Shannon index values for phytoplankton diversity were highest in November during four seasons in coastal waters of the Bohai Sea off Qinhuangdao, China (Dong et al., 2021). Yu et al. (2019) also found that the Shannon and Chao1 index values were both highest in November among seasonal groups in the North China Sea, indicating the highest diversity during the winter. Consistently, seawater samples from the East China Sea had a low Shannon index in spring, and the lowest value was observed in summer (Xu et al., 2015). Gao et al. (2013) also reported that the Shannon index values for the phytoplankton communities in the southern Yellow Sea were lowest in spring. The highest Shannon index value occurred in winter in the subtropical Beibu Gulf area and in autumn in the coastal East China, possibly because the sea temperatures during winter in Beibu Gulf and autumn in higher latitude East China were similar and suitable for phytoplankton growth and reproduction (Xu et al., 2015). In our study, the diversity of the phytoplankton community sharply decreased from winter to spring as the temperature decreased, which might be because some phytoplankton could not grow or survive at relatively low temperatures in the Beibu Gulf area (Yu et al., 2019). These results demonstrate that phytoplankton community structure displays specific characteristics and variations during the seasonal transformation in the Beibu Gulf.

Understanding the relationship between community diversity and stability under environmental perturbations in ecosystems is a crucial issue and is the subject of a long‐standing debate (Guelzow et al., 2017; Loreau & de Mazancourt, 2013). Although there may be a single‐species group that shows unstable dynamic changes in a diverse community, the consensus is that that diversity enhances community stability in ecosystems (McCann, 2000; Ptacnik et al., 2008). For marine phytoplankton, species composition and richness were important for increases of phytoplankton community stability (Corcoran & Boeing, 2012; Guelzow et al., 2017). Thus, our results indicated that higher alpha and beta diversity was beneficial to improving phytoplankton community stability in the Beibu Gulf area, as a more diverse phytoplankton community allowed more variable compositional responses to environmental fluctuations (Allan et al., 2011; Loreau & de Mazancourt, 2013; Vallina et al., 2017).

Ecological stoichiometry is a powerful tool that allowed us to analyze basic biogeochemical patterns and cycling in marine ecosystems (Pujo‐Pay et al., 2011). Our results demonstrate that ES can significantly influence alpha and beta diversity of the phytoplankton community and that it was the main influence of the phytoplankton community assemblage in the subtropical Beibu Gulf. In contrast, He et al. (2013) found that the N:P ratio was negatively correlated with the phytoplankton diversity index in the spring near Weizhou Island in the northern South China Sea. N:P ratios were also negatively correlated with species richness of the entire phytoplankton community in the Cau Hai Lagoon in Vietnam (Nhu et al., 2019). A possible explanation for these inconsistent results is that the positive correlation between the N:P ratio and phytoplankton diversity in our study was based on evaluation of samples collected on a spatio‐temporal scale (different sites, four seasons), whereas the results from the other studies were obtained during one individual season or several months within a short time period. Additionally, the high level of anthropogenic activities in Beibu Gulf intensively accelerates nutrient inputs into the coastal ecosystems, resulting in a eutrophication problem (Li et al., 2020). Therefore, studies that consider different environmental, nutrient, and spatio‐temporal scales in different areas should be conducted. In our study, we also found that ratios of C:N, C:P, N:P, and C:N:P were highest in summer, which may have been because the high growth rate of phytoplankton generated high biomass production under relatively high temperatures and because the massive amount of nutrients in the seawater were absorbed and utilized by the phytoplankton community (Zhou et al., 2021). We also found that the C:N ratio was significantly negatively correlated with AVD, whereas the negative correlations of C:P, N:P, and C:N:P ratios with AVD were not statistically significant. This result suggests that ES rarely impacted the stability of the phytoplankton community in the Beibu Gulf area.

Phytoplankton communities are susceptible to environmental disturbance. Temperature is an important environment factor that influences phytoplankton growth, metabolism, production, and community structure (Dong et al., 2021). Similar to our results, Lv et al. (2014) and Gogoi et al. (2021) both found that temperature was one of the crucial deterministic parameters impacting the phytoplankton community structure. The Netravathi–Gurupura estuary surrounded by several river inlets is similar with our study area and therefore is easily influenced by discharges and effluent nutrients from the rivers (Kumar et al., 2020). In agreement with our results, a significant positive correlation between phytoplankton composition and ${\mathrm{NO}}_{3}^{-}$ content was also observed, indicating that ${\mathrm{NO}}_{3}^{-}$ was an important driver of phytoplankton community structure (Kumar et al., 2020). DIP is frequently the limiting nutrient for phytoplankton growth in marine ecosystems and therefore is a key driver of phytoplankton community variation (Yuan et al., 2018; Zhang et al., 2020). Overall, our results demonstrate that these environmental and nutrient factors are essential to phytoplankton growth, metabolism, and biomass production and therefore could significantly shape the pattern of phytoplankton communities in the subtropical Beibu Gulf.

## 5 CONCLUSIONS

Our results demonstrate that phytoplankton community structure undergoes seasonal variations in the Beibu Gulf. The low water temperatures in winter and spring seem to favor the growth of Bacillariophyceae, whereas the high temperatures in summer and autumn seem to favor Coscinodiscophyceae growth. Furthermore, values of all four of the alpha diversity indices gradually increased from spring to winter, indicating that the seasonal change shifted the community structure. The significantly positive correlations between AVD and Shannon index and Bray–Curtis dissimilarity values demonstrated that a more diverse phytoplankton community conferred more flexible compositional responses to environmental fluctuations. Significant positive associations between Shannon index and Bray–Curtis dissimilarity values and ratios of C:N:P indicated that the nutrient ES of seawater could significantly influence the alpha and beta diversity of the phytoplankton community. Additionally, temperature and NO_3_^−^, DIP, and TDP contents were the main environmental and nutrient drivers of the phytoplankton community assemblages and patterns in the subtropical Beibu Gulf. More studies of the mechanisms underlying the relationships between community stability and diversity and how environmental and nutrient factors influencing the phytoplankton community assemblages are necessary.

## AUTHOR CONTRIBUTIONS

**Qiangsheng Xu:** Conceptualization (equal); formal analysis (equal); methodology (equal); visualization (lead); writing – original draft (lead); writing – review and editing (equal). **Meiqin Huang:** Data curation (equal); investigation (equal); software (equal); validation (equal). **Shu Yang:** Data curation (equal); investigation (equal); validation (equal). **Xiaoli Li:** Data curation (equal); investigation (equal); validation (equal). **Huaxian Zhao:** Investigation (equal); software (equal). **Jinli Tang:** Data curation (equal); investigation (equal). **Gonglingxia Jiang:** Data curation (equal); software (equal). **Zhuoting Li:** Formal analysis (equal); writing – review and editing (equal). **Yuqing Huang:** Conceptualization (equal); methodology (equal). **Ke Dong:** Conceptualization (equal); methodology (equal). **Liangliang Huang:** Conceptualization (equal); methodology (equal). **Nan Li:** Conceptualization (equal); formal analysis (equal); methodology (equal); writing – review and editing (equal).

## CONFLICTS OF INTERESTS

The authors declare that they have no conflicts of interests.

## CONSENT FOR PUBLICATION

All authors consent for publication.

## Supporting information


**Figure S1** Location of sampling sites in the Maowei Sea of the subtropical Beibu Gulf.

**Figure S2** Phytoplankton community compositions at class level for samples collected in spring, summer, autumn, and winter.

**Figure S3** Results of linear regression analysis of the association between AVD and nutrient ES of seawater. (a) AVD vs. C:N; (b) AVD vs. C:P; (c) AVD vs. N:P; (d) AVD vs. C:N:P. Straight lines represent linear relationships, and *p*‐values indicate significant differences.

**Table S1** Physical and biochemical properties of seawater in four seasons of Beibu Gulf.
**Table S2** Number of sequences and OTUs and alpha diversity estimates.

## Data Availability

The datasets presented in this study can be found in NCBI SRA as BioProject PRJNA749375 under access numbers ranging from SAMN20371288 to SAMN20371387.
