# Supplementary figures and images for: Ecological stoichiometry influences phytoplankton alpha and beta diversity rather than the community stability in subtropical bay
Source: Ecol Evol. 2022 Sep 9;12(9):e9301. doi: 10.1002/ece3.9301 (PMC9463046; doi:10.1002/ece3.9301)

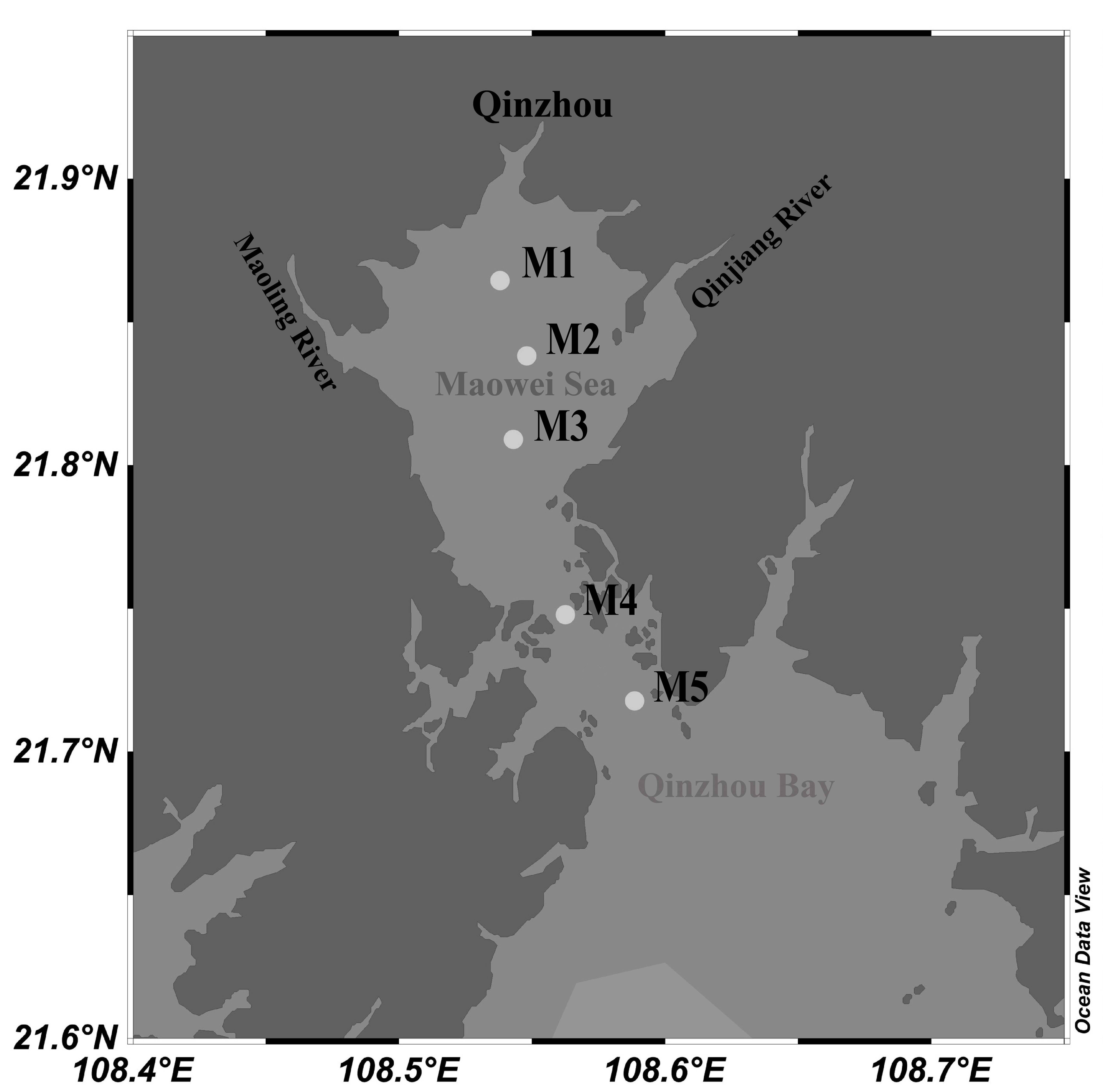

Supplement: Supplementary file 1 — Figure S1 Location of sampling sites in the Maowei Sea of the subtropical Beibu Gulf. [file ECE3-12-e9301-s004.jpg]

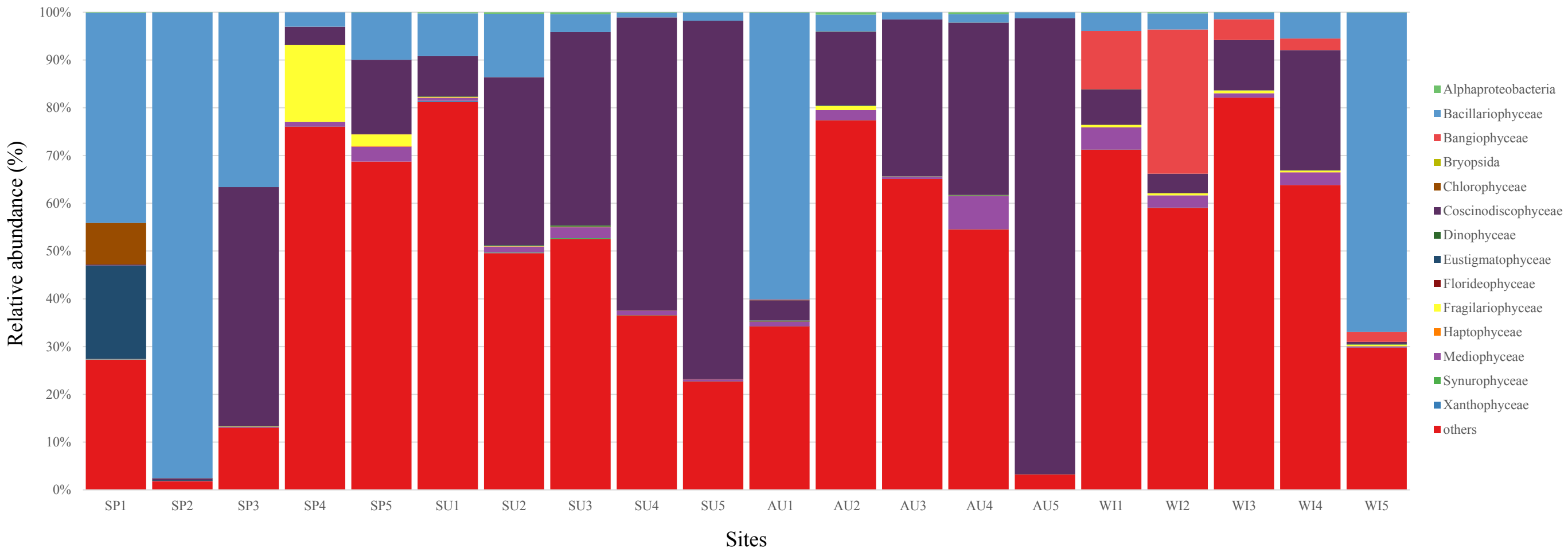

Supplement: Supplementary file 2 — Figure S2 Phytoplankton community compositions at class level for samples collected in spring, summer, autumn, and winter. [file ECE3-12-e9301-s003.pdf]

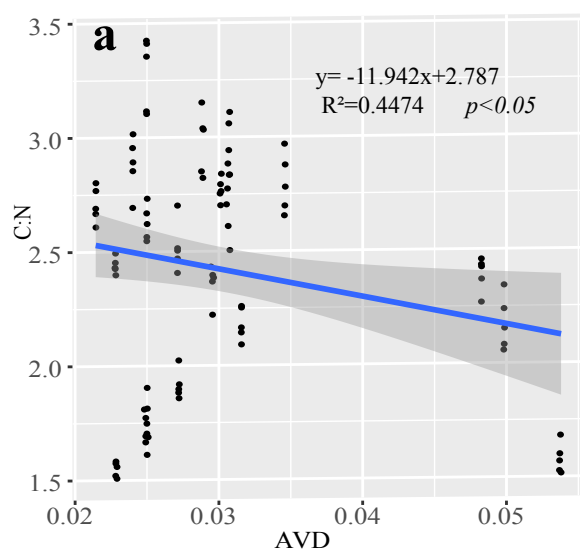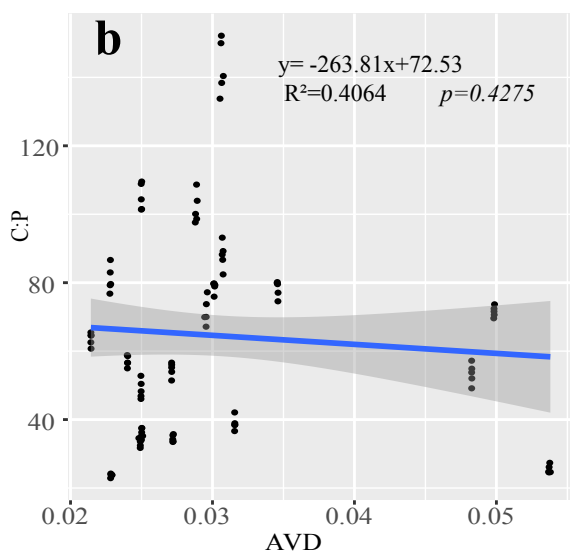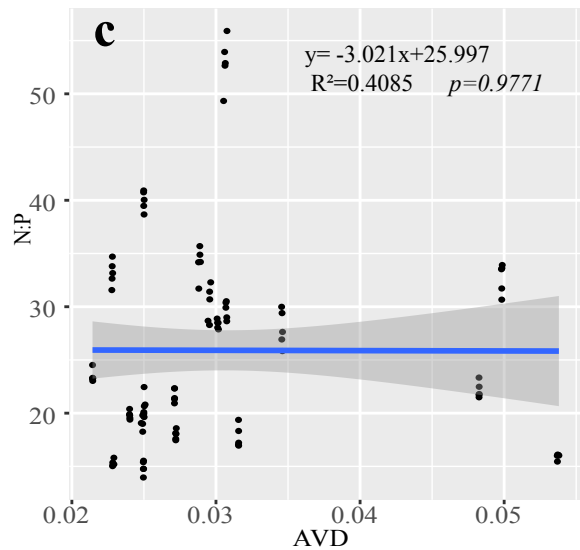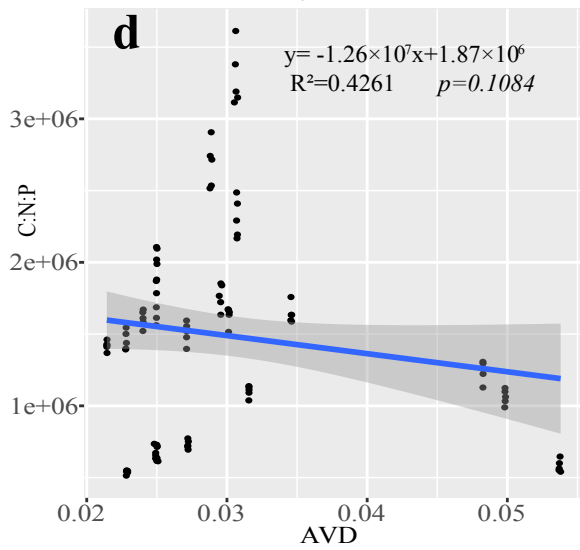

Supplement: Supplementary file 3 — Figure S3 Results of linear regression analysis of the association between AVD and nutrient ES of seawater. (a) AVD vs. C:N; (b) AVD vs. C:P; (c) AVD vs. N:P; (d) AVD vs. C:N:P. Straight lines represent linear relationships, and p‐values indicate significant differences. [file ECE3-12-e9301-s002.pdf]
