# Supplementary material for: Ecological stoichiometry influences phytoplankton alpha and beta diversity rather than the community stability in subtropical bay
Source: Ecol Evol. 2022 Sep 9;12(9):e9301. doi: 10.1002/ece3.9301 (PMC9463046; doi:10.1002/ece3.9301)
Supplement: Supplementary file 4 — Table S1 Physical and biochemical properties of seawater in four seasons of Beibu Gulf. Table S2 Number of sequences and OTUs and alpha diversity estimates. [file ECE3-12-e9301-s001.docx]

Table S1 Physical and biochemical properties of seawater in four seasons of Beibu Gulf.

| **Sites** | **Temperature (°C)** | **pH** | **Salinity (ppt)** | **DO (mg/L)** | **NO_2_^-^  (mg/L)** | **NO_3_^-^ (mg/L)** | **NH_4_^+^(mg/L)** | **Chl-*a(mg/L)*** | **TDN (mg/L)** | **DIN (mg/L)** | **DIP (mg/L)** | **TDP(mg/L)** | **TOC (mg/L)** | **COD (mg/L)** | **C:N** | **C:P** | **N:P** | **C:N:P** |
| --- | --- | --- | --- | --- | --- | --- | --- | --- | --- | --- | --- | --- | --- | --- | --- | --- | --- | --- |
| AU1.1 | 31.92 | 7.24 | 6.02 | 6.59 | 0.055 | 0.416 | 0.225 | 4.56 | 1.030 | 0.695 | 0.041 | 0.065 | 1.82 | 4.13 | 2.36 | 72.40 | 30.66 | 1124370.05 |
| AU1.2 | 31.76 | 7.53 | 6.11 | 6.23 | 0.052 | 0.401 | 0.242 | 4.61 | 1.042 | 0.695 | 0.042 | 0.064 | 1.76 | 4.05 | 2.26 | 71.59 | 31.71 | 1099005.92 |
| AU1.3 | 31.38 | 7.55 | 6.16 | 6.43 | 0.055 | 0.395 | 0.231 | 4.62 | 1.109 | 0.682 | 0.041 | 0.063 | 1.81 | 3.87 | 2.17 | 73.67 | 33.92 | 1062666.04 |
| AU1.4 | 32.96 | 6.94 | 6.18 | 6.27 | 0.052 | 0.407 | 0.234 | 4.25 | 1.125 | 0.693 | 0.041 | 0.065 | 1.75 | 4.01 | 2.08 | 69.59 | 33.52 | 989458.75 |
| AU1.5 | 30.56 | 7.13 | 6.59 | 6.37 | 0.053 | 0.398 | 0.242 | 4.31 | 1.094 | 0.693 | 0.044 | 0.063 | 1.72 | 3.91 | 2.10 | 70.62 | 33.62 | 1032860.01 |
| AU2.1 | 30.21 | 7.05 | 6.70 | 6.36 | 0.041 | 0.408 | 0.157 | 3.74 | 0.836 | 0.606 | 0.039 | 0.040 | 1.72 | 3.87 | 2.74 | 109.55 | 40.04 | 2095833.26 |
| AU2.2 | 32.65 | 7.72 | 7.18 | 6.36 | 0.041 | 0.398 | 0.160 | 3.86 | 0.817 | 0.599 | 0.037 | 0.041 | 1.61 | 3.87 | 2.63 | 101.51 | 38.67 | 1988894.99 |
| AU2.3 | 32.71 | 7.46 | 6.56 | 6.28 | 0.043 | 0.434 | 0.158 | 3.74 | 0.827 | 0.635 | 0.038 | 0.039 | 1.58 | 4.07 | 2.55 | 104.35 | 40.92 | 2019149.98 |
| AU2.4 | 30.12 | 7.40 | 7.16 | 6.29 | 0.044 | 0.420 | 0.152 | 3.73 | 0.863 | 0.616 | 0.040 | 0.042 | 1.66 | 3.76 | 2.57 | 101.37 | 39.47 | 1879744.68 |
| AU2.5 | 32.59 | 7.15 | 7.00 | 6.38 | 0.044 | 0.427 | 0.150 | 3.62 | 0.827 | 0.621 | 0.041 | 0.039 | 1.66 | 3.88 | 2.67 | 108.87 | 40.75 | 2106576.26 |
| AU3.1 | 30.72 | 7.29 | 8.87 | 6.89 | 0.092 | 0.368 | 0.060 | 2.81 | 0.907 | 0.520 | 0.037 | 0.054 | 1.65 | 3.56 | 2.43 | 79.28 | 32.64 | 1398799.51 |
| AU3.2 | 30.98 | 7.48 | 8.95 | 6.65 | 0.087 | 0.366 | 0.055 | 2.81 | 0.885 | 0.508 | 0.038 | 0.052 | 1.59 | 3.47 | 2.40 | 79.61 | 33.16 | 1438558.82 |
| AU3.3 | 31.94 | 7.72 | 9.73 | 6.85 | 0.093 | 0.366 | 0.061 | 2.80 | 0.882 | 0.520 | 0.038 | 0.054 | 1.61 | 3.70 | 2.43 | 76.79 | 31.56 | 1393253.02 |
| AU3.4 | 32.29 | 7.29 | 8.99 | 6.75 | 0.093 | 0.370 | 0.061 | 2.89 | 0.885 | 0.523 | 0.036 | 0.051 | 1.63 | 3.60 | 2.46 | 82.99 | 33.80 | 1501130.07 |
| AU3.5 | 32.23 | 7.44 | 9.34 | 6.91 | 0.087 | 0.364 | 0.056 | 2.79 | 0.897 | 0.507 | 0.040 | 0.050 | 1.68 | 3.51 | 2.50 | 86.63 | 34.71 | 1545302.12 |
| AU4.1 | 31.93 | 7.18 | 11.20 | 6.26 | 0.069 | 0.346 | 0.066 | 2.31 | 0.779 | 0.480 | 0.041 | 0.051 | 1.58 | 3.12 | 2.71 | 79.58 | 29.40 | 1633970.12 |
| AU4.2 | 31.97 | 7.26 | 11.20 | 6.23 | 0.067 | 0.336 | 0.067 | 2.10 | 0.752 | 0.469 | 0.043 | 0.056 | 1.63 | 2.97 | 2.89 | 74.56 | 25.83 | 1587090.33 |
| AU4.3 | 31.84 | 7.51 | 11.27 | 6.59 | 0.067 | 0.346 | 0.065 | 2.13 | 0.755 | 0.478 | 0.044 | 0.053 | 1.58 | 3.07 | 2.79 | 77.07 | 27.63 | 1633848.37 |
| AU4.4 | 31.02 | 7.19 | 10.86 | 6.24 | 0.066 | 0.333 | 0.063 | 2.27 | 0.729 | 0.462 | 0.042 | 0.052 | 1.63 | 2.89 | 2.98 | 80.16 | 26.93 | 1758373.87 |
| AU4.5 | 30.52 | 7.66 | 10.91 | 6.80 | 0.065 | 0.340 | 0.069 | 2.13 | 0.801 | 0.474 | 0.043 | 0.052 | 1.60 | 3.15 | 2.66 | 79.89 | 29.99 | 1596210.22 |
| AU5.1 | 31.33 | 7.53 | 15.56 | 5.62 | 0.076 | 0.263 | 0.106 | 2.41 | 0.733 | 0.446 | 0.041 | 0.058 | 1.47 | 2.82 | 2.67 | 65.45 | 24.52 | 1428030.50 |
| AU5.2 | 32.83 | 7.29 | 15.31 | 6.09 | 0.074 | 0.259 | 0.104 | 2.38 | 0.710 | 0.437 | 0.044 | 0.059 | 1.39 | 2.73 | 2.61 | 60.72 | 23.26 | 1368623.84 |
| AU5.3 | 30.78 | 7.23 | 15.53 | 5.63 | 0.070 | 0.266 | 0.102 | 2.54 | 0.707 | 0.439 | 0.042 | 0.059 | 1.49 | 2.93 | 2.80 | 64.59 | 23.03 | 1461588.84 |
| AU5.4 | 32.57 | 7.62 | 15.21 | 5.55 | 0.070 | 0.274 | 0.103 | 2.49 | 0.727 | 0.446 | 0.044 | 0.060 | 1.51 | 2.95 | 2.77 | 64.57 | 23.31 | 1421159.04 |
| AU5.5 | 32.31 | 7.62 | 15.21 | 5.66 | 0.075 | 0.274 | 0.100 | 2.50 | 0.709 | 0.448 | 0.045 | 0.059 | 1.43 | 2.77 | 2.69 | 62.56 | 23.24 | 1411513.05 |
| SP1.1 | 24.24 | 8.02 | 19.71 | 9.39 | 0.022 | 0.447 | 0.168 | 1.61 | 0.884 | 0.637 | 0.039 | 0.086 | 1.13 | 2.97 | 1.71 | 34.07 | 19.95 | 616534.63 |
| SP1.2 | 24.52 | 7.60 | 18.26 | 9.10 | 0.021 | 0.425 | 0.169 | 1.65 | 0.839 | 0.615 | 0.037 | 0.083 | 1.20 | 2.96 | 1.91 | 37.46 | 19.64 | 714099.45 |
| SP1.3 | 24.33 | 7.43 | 18.96 | 8.95 | 0.022 | 0.456 | 0.172 | 1.58 | 0.836 | 0.650 | 0.035 | 0.078 | 1.14 | 3.14 | 1.82 | 37.54 | 20.66 | 718568.58 |
| SP1.4 | 23.04 | 7.60 | 19.50 | 8.85 | 0.021 | 0.465 | 0.177 | 1.66 | 0.912 | 0.663 | 0.035 | 0.079 | 1.10 | 3.20 | 1.61 | 36.25 | 22.45 | 635867.95 |
| SP1.5 | 22.82 | 8.00 | 19.52 | 9.53 | 0.021 | 0.434 | 0.183 | 1.64 | 0.918 | 0.638 | 0.038 | 0.085 | 1.16 | 3.13 | 1.69 | 35.20 | 20.81 | 613884.09 |
| SP2.1 | 24.60 | 7.78 | 18.58 | 8.53 | 0.021 | 0.430 | 0.173 | 1.65 | 0.820 | 0.624 | 0.047 | 0.080 | 1.04 | 2.71 | 1.70 | 33.59 | 19.80 | 655064.43 |
| SP2.2 | 24.82 | 7.45 | 18.24 | 8.79 | 0.022 | 0.425 | 0.170 | 1.61 | 0.773 | 0.617 | 0.047 | 0.074 | 1.01 | 2.74 | 1.75 | 35.19 | 20.10 | 728829.70 |
| SP2.3 | 23.93 | 8.00 | 19.79 | 9.03 | 0.023 | 0.451 | 0.182 | 1.58 | 0.752 | 0.655 | 0.048 | 0.076 | 1.02 | 2.84 | 1.81 | 34.57 | 19.07 | 735315.23 |
| SP2.4 | 23.24 | 7.75 | 19.16 | 8.74 | 0.022 | 0.444 | 0.181 | 1.56 | 0.772 | 0.647 | 0.046 | 0.082 | 1.03 | 2.84 | 1.78 | 32.43 | 18.26 | 672255.43 |
| SP2.5 | 23.57 | 7.88 | 18.07 | 9.25 | 0.021 | 0.437 | 0.185 | 1.58 | 0.801 | 0.643 | 0.046 | 0.082 | 1.00 | 2.85 | 1.67 | 31.71 | 19.00 | 633656.87 |
| SP3.1 | 23.95 | 7.55 | 20.16 | 9.64 | 0.019 | 0.381 | 0.162 | 1.32 | 0.761 | 0.562 | 0.068 | 0.079 | 1.10 | 3.05 | 1.92 | 35.74 | 18.58 | 751890.71 |
| SP3.2 | 24.55 | 7.84 | 18.95 | 8.85 | 0.018 | 0.378 | 0.163 | 1.25 | 0.741 | 0.559 | 0.068 | 0.082 | 1.06 | 3.05 | 1.90 | 33.46 | 17.59 | 722971.04 |
| SP3.3 | 23.19 | 7.85 | 20.12 | 8.85 | 0.020 | 0.362 | 0.172 | 1.30 | 0.774 | 0.553 | 0.070 | 0.083 | 1.08 | 2.88 | 1.86 | 33.63 | 18.05 | 694768.22 |
| SP3.4 | 23.47 | 7.98 | 20.45 | 9.39 | 0.019 | 0.382 | 0.176 | 1.24 | 0.764 | 0.577 | 0.065 | 0.082 | 1.08 | 2.87 | 1.89 | 34.19 | 18.12 | 715713.53 |
| SP3.5 | 24.93 | 7.49 | 20.00 | 9.37 | 0.019 | 0.371 | 0.169 | 1.31 | 0.733 | 0.560 | 0.068 | 0.081 | 1.11 | 2.87 | 2.03 | 35.41 | 17.46 | 773025.76 |
| SP4.1 | 23.97 | 7.88 | 21.97 | 9.42 | 0.012 | 0.344 | 0.123 | 0.93 | 0.677 | 0.480 | 0.028 | 0.082 | 0.86 | 2.13 | 1.70 | 27.36 | 16.06 | 646735.72 |
| SP4.2 | 23.66 | 7.59 | 22.12 | 8.67 | 0.013 | 0.343 | 0.122 | 0.89 | 0.696 | 0.478 | 0.030 | 0.084 | 0.85 | 2.16 | 1.62 | 26.11 | 16.10 | 600279.48 |
| SP4.3 | 22.97 | 8.12 | 21.49 | 8.93 | 0.013 | 0.352 | 0.120 | 0.93 | 0.729 | 0.485 | 0.030 | 0.088 | 0.84 | 2.22 | 1.54 | 24.64 | 16.02 | 541030.74 |
| SP4.4 | 24.57 | 8.06 | 22.01 | 8.91 | 0.013 | 0.355 | 0.112 | 0.89 | 0.698 | 0.480 | 0.028 | 0.088 | 0.83 | 2.26 | 1.59 | 24.58 | 15.46 | 563261.99 |
| SP4.5 | 25.15 | 7.82 | 22.08 | 8.58 | 0.013 | 0.339 | 0.120 | 0.88 | 0.718 | 0.472 | 0.028 | 0.087 | 0.83 | 2.14 | 1.55 | 24.77 | 16.00 | 551922.05 |
| SP5.1 | 24.56 | 7.37 | 23.58 | 8.91 | 0.012 | 0.333 | 0.117 | 1.64 | 0.714 | 0.462 | 0.039 | 0.090 | 0.84 | 2.22 | 1.58 | 24.21 | 15.36 | 542564.42 |
| SP5.2 | 24.99 | 7.97 | 22.53 | 8.59 | 0.012 | 0.351 | 0.118 | 1.60 | 0.713 | 0.482 | 0.039 | 0.087 | 0.81 | 2.19 | 1.51 | 23.87 | 15.81 | 535627.93 |
| SP5.3 | 23.34 | 7.44 | 22.23 | 8.44 | 0.012 | 0.326 | 0.114 | 1.56 | 0.695 | 0.452 | 0.038 | 0.089 | 0.81 | 2.20 | 1.56 | 23.73 | 15.20 | 546680.33 |
| SP5.4 | 24.56 | 7.78 | 22.96 | 8.88 | 0.013 | 0.331 | 0.118 | 1.66 | 0.697 | 0.462 | 0.040 | 0.089 | 0.83 | 2.18 | 1.59 | 23.95 | 15.11 | 549969.38 |
| SP5.5 | 24.65 | 7.70 | 22.50 | 8.42 | 0.012 | 0.352 | 0.118 | 1.56 | 0.712 | 0.482 | 0.039 | 0.092 | 0.81 | 2.11 | 1.52 | 22.88 | 15.03 | 513837.13 |
| SU1.1 | 27.34 | 7.28 | 11.63 | 6.56 | 0.016 | 0.692 | 0.066 | 5.25 | 0.762 | 0.773 | 0.013 | 0.051 | 1.58 | 4.29 | 2.76 | 79.69 | 28.88 | 1672314.30 |
| SU1.2 | 29.55 | 7.75 | 11.87 | 6.29 | 0.016 | 0.701 | 0.063 | 5.34 | 0.772 | 0.779 | 0.013 | 0.053 | 1.60 | 4.04 | 2.77 | 78.90 | 28.49 | 1634472.23 |
| SU1.3 | 27.37 | 7.48 | 11.37 | 6.59 | 0.014 | 0.707 | 0.063 | 5.68 | 0.768 | 0.784 | 0.013 | 0.053 | 1.64 | 3.96 | 2.85 | 79.31 | 27.87 | 1652807.09 |
| SU1.4 | 29.60 | 7.73 | 10.96 | 6.57 | 0.015 | 0.695 | 0.063 | 5.32 | 0.802 | 0.773 | 0.012 | 0.055 | 1.63 | 4.36 | 2.71 | 75.92 | 28.04 | 1515463.09 |
| SU1.5 | 29.15 | 7.68 | 11.03 | 6.66 | 0.016 | 0.664 | 0.066 | 5.50 | 0.765 | 0.745 | 0.013 | 0.052 | 1.61 | 4.25 | 2.80 | 79.78 | 28.50 | 1669318.38 |
| SU2.1 | 27.29 | 7.41 | 13.40 | 6.67 | 0.015 | 0.607 | 0.066 | 3.97 | 0.674 | 0.687 | 0.019 | 0.025 | 1.46 | 3.81 | 2.89 | 152.17 | 52.65 | 3612238.00 |
| SU2.2 | 27.66 | 7.94 | 13.68 | 7.06 | 0.015 | 0.579 | 0.067 | 3.72 | 0.687 | 0.661 | 0.019 | 0.027 | 1.40 | 3.71 | 2.71 | 133.77 | 49.33 | 3114675.73 |
| SU2.3 | 29.45 | 8.02 | 14.27 | 6.79 | 0.015 | 0.616 | 0.069 | 3.94 | 0.694 | 0.699 | 0.020 | 0.025 | 1.36 | 3.71 | 2.62 | 138.38 | 52.89 | 3190071.34 |
| SU2.4 | 27.01 | 7.92 | 14.32 | 6.60 | 0.015 | 0.599 | 0.072 | 3.89 | 0.710 | 0.687 | 0.019 | 0.026 | 1.48 | 3.85 | 2.78 | 149.98 | 53.93 | 3379932.79 |
| SU2.5 | 28.63 | 7.40 | 14.59 | 6.62 | 0.015 | 0.625 | 0.070 | 3.89 | 0.713 | 0.710 | 0.019 | 0.025 | 1.34 | 3.84 | 2.51 | 140.39 | 55.91 | 3148505.71 |
| SU3.1 | 27.88 | 7.42 | 17.17 | 6.96 | 0.011 | 0.573 | 0.109 | 2.72 | 0.612 | 0.693 | 0.012 | 0.035 | 1.40 | 3.21 | 3.04 | 103.93 | 34.20 | 2716193.50 |
| SU3.2 | 27.08 | 8.14 | 17.30 | 7.19 | 0.011 | 0.577 | 0.107 | 2.57 | 0.623 | 0.695 | 0.013 | 0.035 | 1.32 | 3.37 | 2.83 | 98.64 | 34.89 | 2534793.93 |
| SU3.3 | 28.07 | 7.89 | 17.60 | 6.78 | 0.011 | 0.587 | 0.109 | 2.62 | 0.598 | 0.707 | 0.014 | 0.032 | 1.37 | 3.38 | 3.04 | 108.66 | 35.69 | 2906531.20 |
| SU3.4 | 28.35 | 7.68 | 18.22 | 6.99 | 0.011 | 0.604 | 0.103 | 2.76 | 0.621 | 0.718 | 0.013 | 0.035 | 1.33 | 3.40 | 2.86 | 97.61 | 34.18 | 2515799.76 |
| SU3.5 | 27.77 | 7.70 | 17.60 | 6.83 | 0.011 | 0.568 | 0.103 | 2.74 | 0.584 | 0.682 | 0.013 | 0.036 | 1.38 | 3.51 | 3.16 | 100.09 | 31.70 | 2741662.62 |
| SU4.1 | 28.58 | 7.80 | 18.26 | 6.92 | 0.009 | 0.529 | 0.139 | 2.04 | 0.616 | 0.677 | 0.012 | 0.040 | 1.36 | 3.34 | 2.95 | 88.22 | 29.91 | 2291304.65 |
| SU4.2 | 27.05 | 7.51 | 19.53 | 6.85 | 0.009 | 0.555 | 0.142 | 2.04 | 0.599 | 0.706 | 0.013 | 0.038 | 1.38 | 3.35 | 3.07 | 93.16 | 30.38 | 2486880.46 |
| SU4.3 | 26.88 | 8.15 | 19.49 | 6.41 | 0.009 | 0.527 | 0.139 | 1.94 | 0.593 | 0.675 | 0.012 | 0.040 | 1.38 | 3.26 | 3.12 | 89.24 | 28.64 | 2409387.17 |
| SU4.4 | 28.13 | 7.83 | 18.72 | 6.63 | 0.009 | 0.530 | 0.133 | 2.01 | 0.601 | 0.671 | 0.011 | 0.040 | 1.28 | 3.22 | 2.84 | 82.42 | 29.00 | 2193468.55 |
| SU4.5 | 27.94 | 7.49 | 19.93 | 6.71 | 0.009 | 0.516 | 0.136 | 1.91 | 0.640 | 0.661 | 0.012 | 0.041 | 1.36 | 3.44 | 2.84 | 86.69 | 30.51 | 2167190.33 |
| SU5.1 | 28.16 | 7.86 | 21.89 | 5.91 | 0.006 | 0.445 | 0.161 | 1.95 | 0.633 | 0.612 | 0.013 | 0.043 | 1.16 | 2.83 | 2.44 | 69.94 | 28.67 | 1766572.89 |
| SU5.2 | 27.11 | 7.86 | 22.70 | 6.20 | 0.006 | 0.432 | 0.160 | 1.93 | 0.624 | 0.598 | 0.014 | 0.043 | 1.11 | 2.88 | 2.37 | 67.14 | 28.29 | 1722639.95 |
| SU5.3 | 27.69 | 8.09 | 22.03 | 5.91 | 0.006 | 0.441 | 0.161 | 2.08 | 0.685 | 0.609 | 0.013 | 0.042 | 1.14 | 2.80 | 2.23 | 69.99 | 31.41 | 1634865.28 |
| SU5.4 | 26.80 | 7.47 | 21.40 | 5.96 | 0.006 | 0.437 | 0.155 | 1.99 | 0.637 | 0.598 | 0.013 | 0.040 | 1.15 | 2.81 | 2.40 | 73.72 | 30.69 | 1852736.25 |
| SU5.5 | 27.61 | 7.69 | 22.86 | 6.09 | 0.006 | 0.442 | 0.161 | 2.08 | 0.671 | 0.610 | 0.014 | 0.040 | 1.20 | 3.08 | 2.39 | 77.20 | 32.29 | 1839993.78 |
| WI1.1 | 14.87 | 7.50 | 19.42 | 8.55 | 0.010 | 0.229 | 0.072 | 1.14 | 0.677 | 0.311 | 0.027 | 0.058 | 1.24 | 2.92 | 2.44 | 54.86 | 22.48 | 1295998.80 |
| WI1.2 | 15.61 | 7.24 | 18.34 | 8.86 | 0.010 | 0.210 | 0.078 | 1.09 | 0.700 | 0.297 | 0.029 | 0.058 | 1.29 | 3.05 | 2.45 | 57.20 | 23.34 | 1306670.37 |
| WI1.3 | 14.18 | 7.50 | 19.03 | 8.37 | 0.010 | 0.212 | 0.074 | 1.09 | 0.697 | 0.297 | 0.028 | 0.063 | 1.19 | 3.10 | 2.29 | 49.13 | 21.49 | 1127839.27 |
| WI1.4 | 15.13 | 7.08 | 18.21 | 8.87 | 0.010 | 0.221 | 0.077 | 1.12 | 0.684 | 0.308 | 0.027 | 0.061 | 1.27 | 2.91 | 2.47 | 53.77 | 21.73 | 1257598.36 |
| WI1.5 | 15.47 | 7.52 | 19.34 | 8.78 | 0.010 | 0.218 | 0.077 | 1.11 | 0.681 | 0.304 | 0.027 | 0.061 | 1.22 | 2.98 | 2.39 | 52.02 | 21.80 | 1222791.51 |
| WI2.1 | 14.76 | 7.17 | 18.01 | 8.70 | 0.007 | 0.140 | 0.071 | 1.25 | 0.452 | 0.219 | 0.024 | 0.057 | 1.16 | 2.21 | 3.43 | 52.78 | 15.40 | 1870216.61 |
| WI2.2 | 14.65 | 7.34 | 19.23 | 8.93 | 0.007 | 0.144 | 0.071 | 1.35 | 0.452 | 0.222 | 0.024 | 0.059 | 1.16 | 2.24 | 3.41 | 50.43 | 14.77 | 1785061.61 |
| WI2.3 | 14.49 | 7.12 | 19.51 | 8.90 | 0.007 | 0.154 | 0.065 | 1.24 | 0.472 | 0.226 | 0.024 | 0.062 | 1.10 | 2.36 | 3.12 | 46.04 | 14.76 | 1561979.47 |
| WI2.4 | 15.07 | 7.07 | 19.19 | 8.54 | 0.007 | 0.141 | 0.070 | 1.27 | 0.479 | 0.218 | 0.025 | 0.060 | 1.12 | 2.30 | 3.11 | 48.25 | 15.53 | 1613148.81 |
| WI2.5 | 14.27 | 7.50 | 19.66 | 8.91 | 0.007 | 0.147 | 0.066 | 1.35 | 0.445 | 0.220 | 0.024 | 0.062 | 1.12 | 2.31 | 3.36 | 46.87 | 13.96 | 1686846.59 |
| WI3.1 | 15.22 | 7.60 | 19.45 | 9.08 | 0.007 | 0.167 | 0.072 | 1.47 | 0.561 | 0.246 | 0.020 | 0.056 | 1.27 | 2.76 | 3.02 | 58.55 | 19.40 | 1670835.78 |
| WI3.2 | 14.73 | 7.80 | 18.80 | 8.86 | 0.007 | 0.175 | 0.074 | 1.45 | 0.570 | 0.256 | 0.020 | 0.056 | 1.26 | 2.60 | 2.96 | 58.84 | 19.89 | 1651784.72 |
| WI3.3 | 14.43 | 7.80 | 18.70 | 8.91 | 0.007 | 0.161 | 0.072 | 1.49 | 0.572 | 0.239 | 0.020 | 0.057 | 1.24 | 2.74 | 2.90 | 56.61 | 19.55 | 1582872.17 |
| WI3.4 | 15.13 | 7.76 | 18.91 | 9.15 | 0.007 | 0.167 | 0.072 | 1.53 | 0.578 | 0.246 | 0.020 | 0.055 | 1.17 | 2.60 | 2.70 | 54.97 | 20.39 | 1521701.03 |
| WI3.5 | 15.41 | 7.87 | 18.59 | 8.71 | 0.007 | 0.167 | 0.072 | 1.43 | 0.562 | 0.245 | 0.021 | 0.055 | 1.20 | 2.67 | 2.86 | 56.57 | 19.80 | 1610999.17 |
| WI4.1 | 15.16 | 7.18 | 21.45 | 8.91 | 0.005 | 0.120 | 0.051 | 0.78 | 0.577 | 0.176 | 0.016 | 0.050 | 1.08 | 1.87 | 2.51 | 56.03 | 22.33 | 1554705.60 |
| WI4.2 | 15.07 | 7.12 | 21.92 | 8.54 | 0.005 | 0.119 | 0.047 | 0.78 | 0.568 | 0.171 | 0.017 | 0.053 | 1.15 | 1.79 | 2.71 | 56.63 | 20.93 | 1594092.07 |
| WI4.3 | 14.68 | 7.78 | 20.58 | 8.77 | 0.005 | 0.111 | 0.048 | 0.83 | 0.568 | 0.164 | 0.016 | 0.051 | 1.07 | 1.81 | 2.52 | 53.94 | 21.41 | 1520108.81 |
| WI4.4 | 15.23 | 7.79 | 20.82 | 8.83 | 0.005 | 0.120 | 0.051 | 0.82 | 0.598 | 0.176 | 0.017 | 0.052 | 1.11 | 1.78 | 2.47 | 55.22 | 22.32 | 1477887.13 |
| WI4.5 | 15.68 | 7.50 | 21.84 | 9.04 | 0.005 | 0.118 | 0.051 | 0.81 | 0.589 | 0.174 | 0.018 | 0.054 | 1.07 | 1.86 | 2.41 | 51.42 | 21.33 | 1396843.76 |
| WI5.1 | 14.47 | 7.74 | 22.43 | 9.63 | 0.005 | 0.131 | 0.067 | 1.27 | 0.592 | 0.202 | 0.014 | 0.059 | 0.96 | 1.87 | 2.17 | 42.08 | 19.37 | 1137469.61 |
| WI5.2 | 14.83 | 7.43 | 21.47 | 9.18 | 0.005 | 0.135 | 0.065 | 1.27 | 0.564 | 0.205 | 0.014 | 0.060 | 0.89 | 1.88 | 2.10 | 38.46 | 18.33 | 1091794.74 |
| WI5.3 | 14.21 | 7.25 | 21.63 | 9.29 | 0.005 | 0.127 | 0.065 | 1.37 | 0.551 | 0.197 | 0.014 | 0.062 | 0.93 | 1.78 | 2.26 | 38.92 | 17.22 | 1131081.30 |
| WI5.4 | 15.44 | 7.48 | 22.46 | 9.11 | 0.005 | 0.124 | 0.063 | 1.25 | 0.565 | 0.192 | 0.014 | 0.064 | 0.91 | 1.87 | 2.15 | 36.64 | 17.03 | 1038067.14 |
| WI5.5 | 15.38 | 7.73 | 21.63 | 9.42 | 0.005 | 0.123 | 0.066 | 1.26 | 0.552 | 0.194 | 0.014 | 0.063 | 0.94 | 1.82 | 2.27 | 38.42 | 16.95 | 1113119.01 |

Table S2 Number of sequences and OTUs and alpha diversity estimates.

| **Seasons** | **Sites** | **OTUs** | **No. of Seq** | **Shannon** | **Simpson** | **Chao1** | **Coverage(%)** |
| --- | --- | --- | --- | --- | --- | --- | --- |
| Spring | SP1.1 | 124 | 8093 | 2.4495 | 0.8198 | 172.2353 | 99.4934 |
|  | SP1.2 | 126 | 8081 | 2.4381 | 0.8181 | 179.1176 | 99.4679 |
|  | SP1.3 | 124 | 8080 | 2.4430 | 0.8194 | 185.5000 | 99.4802 |
|  | SP1.4 | 126 | 8083 | 2.4436 | 0.8193 | 179.1176 | 99.4680 |
|  | SP1.5 | 125 | 8052 | 2.4358 | 0.8173 | 158.4762 | 99.5281 |
|  | SP2.1 | 192 | 19403 | 1.1237 | 0.2980 | 318.0667 | 99.6805 |
|  | SP2.2 | 195 | 19392 | 1.1237 | 0.2980 | 311.3684 | 99.6545 |
|  | SP2.3 | 195 | 19454 | 1.1262 | 0.2987 | 329.0625 | 99.6607 |
|  | SP2.4 | 197 | 19408 | 1.1255 | 0.2979 | 335.1875 | 99.6548 |
|  | SP2.5 | 197 | 19411 | 1.1331 | 0.3011 | 323.5556 | 99.6497 |
|  | SP3.1 | 144 | 21196 | 2.1779 | 0.6732 | 198.0909 | 99.8349 |
|  | SP3.2 | 139 | 21218 | 2.1856 | 0.6748 | 175.9091 | 99.8633 |
|  | SP3.3 | 143 | 21181 | 2.1848 | 0.6743 | 183.6154 | 99.8442 |
|  | SP3.4 | 146 | 21211 | 2.1888 | 0.6745 | 198.5000 | 99.8303 |
|  | SP3.5 | 141 | 21215 | 2.1864 | 0.6742 | 190.6000 | 99.8492 |
|  | SP4.1 | 343 | 51191 | 2.7104 | 0.7532 | 493.0556 | 99.8554 |
|  | SP4.2 | 340 | 51212 | 2.7086 | 0.7531 | 474.5263 | 99.8594 |
|  | SP4.3 | 341 | 51180 | 2.7072 | 0.7526 | 468.8000 | 99.8593 |
|  | SP4.4 | 342 | 51202 | 2.7090 | 0.7532 | 473.4000 | 99.8574 |
|  | SP4.5 | 339 | 51200 | 2.7088 | 0.7529 | 514.2000 | 99.8574 |
|  | SP5.1 | 202 | 11433 | 3.1357 | 0.9064 | 249.9032 | 99.5189 |
|  | SP5.2 | 206 | 11428 | 3.1462 | 0.9075 | 265.0323 | 99.4662 |
|  | SP5.3 | 202 | 11431 | 3.1414 | 0.9075 | 245.0625 | 99.5363 |
|  | SP5.4 | 203 | 11453 | 3.1397 | 0.9067 | 251.1250 | 99.5110 |
|  | SP5.5 | 205 | 11452 | 3.1419 | 0.9070 | 256.8485 | 99.4848 |
| Summer | SU1.1 | 110 | 3261 | 2.1862 | 0.7332 | 147.0500 | 98.8040 |
|  | SU1.2 | 107 | 3219 | 2.1920 | 0.7348 | 146.3750 | 98.8816 |
|  | SU1.3 | 108 | 3222 | 2.1830 | 0.7332 | 145.0000 | 98.8516 |
|  | SU1.4 | 106 | 3244 | 2.1715 | 0.7306 | 145.3750 | 98.8903 |
|  | SU1.5 | 108 | 3240 | 2.1729 | 0.7304 | 147.1765 | 98.8580 |
|  | SU2.1 | 170 | 1656 | 3.5082 | 0.8891 | 264.5455 | 96.0749 |
|  | SU2.2 | 180 | 1674 | 3.5573 | 0.8898 | 259.4444 | 96.0573 |
|  | SU2.3 | 175 | 1660 | 3.5337 | 0.8901 | 261.6667 | 96.0843 |
|  | SU2.4 | 169 | 1651 | 3.5352 | 0.8885 | 237.4400 | 96.4264 |
|  | SU2.5 | 167 | 1648 | 3.5060 | 0.8878 | 241.3913 | 96.4199 |
|  | SU3.1 | 187 | 2605 | 3.6370 | 0.9066 | 260.5000 | 98.1190 |
|  | SU3.2 | 192 | 2617 | 3.6511 | 0.9056 | 233.5769 | 98.2041 |
|  | SU3.3 | 189 | 2615 | 3.6512 | 0.9063 | 238.1364 | 98.2027 |
|  | SU3.4 | 190 | 2644 | 3.6159 | 0.9031 | 259.7895 | 98.0333 |
|  | SU3.5 | 192 | 2615 | 3.6777 | 0.9098 | 228.6667 | 98.2792 |
|  | SU4.1 | 187 | 6767 | 3.1757 | 0.8237 | 210.2143 | 99.6158 |
|  | SU4.2 | 187 | 6755 | 3.1851 | 0.8250 | 214.0833 | 99.6151 |
|  | SU4.3 | 187 | 6800 | 3.1917 | 0.8252 | 234.2500 | 99.5882 |
|  | SU4.4 | 186 | 6772 | 3.1770 | 0.8231 | 225.0000 | 99.6013 |
|  | SU4.5 | 188 | 6791 | 3.1690 | 0.8224 | 215.0833 | 99.6171 |
|  | SU5.1 | 186 | 10170 | 2.2144 | 0.5930 | 209.2143 | 99.7443 |
|  | SU5.2 | 186 | 10130 | 2.2259 | 0.5953 | 213.0000 | 99.7236 |
|  | SU5.3 | 186 | 10140 | 2.2147 | 0.5934 | 229.5000 | 99.7041 |
|  | SU5.4 | 187 | 10148 | 2.2164 | 0.5932 | 223.9091 | 99.7142 |
|  | SU5.5 | 185 | 10114 | 2.2103 | 0.5929 | 210.0000 | 99.7429 |
| Autumn | AU1.1 | 846 | 80949 | 4.0254 | 0.8952 | 951.2083 | 99.8752 |
|  | AU1.2 | 847 | 80926 | 4.0243 | 0.8951 | 950.0612 | 99.8752 |
|  | AU1.3 | 847 | 80911 | 4.0240 | 0.8949 | 946.0000 | 99.8764 |
|  | AU1.4 | 847 | 80945 | 4.0232 | 0.8950 | 950.0612 | 99.8752 |
|  | AU1.5 | 847 | 80925 | 4.0251 | 0.8951 | 954.3125 | 99.8740 |
|  | AU2.1 | 304 | 2086 | 3.5683 | 0.8111 | 514.1224 | 93.0968 |
|  | AU2.2 | 301 | 2060 | 3.5791 | 0.8124 | 491.1224 | 93.3495 |
|  | AU2.3 | 309 | 2081 | 3.5390 | 0.8061 | 531.0588 | 92.7439 |
|  | AU2.4 | 303 | 2115 | 3.5775 | 0.8137 | 513.0000 | 93.3333 |
|  | AU2.5 | 295 | 2096 | 3.5482 | 0.8088 | 451.3077 | 93.8931 |
|  | AU3.1 | 170 | 2806 | 3.4886 | 0.8987 | 267.5000 | 97.6479 |
|  | AU3.2 | 176 | 2808 | 3.4506 | 0.8907 | 376.2000 | 97.2222 |
|  | AU3.3 | 172 | 2842 | 3.4570 | 0.8935 | 265.8400 | 97.5721 |
|  | AU3.4 | 173 | 2840 | 3.4700 | 0.8946 | 258.8000 | 97.6761 |
|  | AU3.5 | 176 | 2818 | 3.4449 | 0.8895 | 287.1304 | 97.4450 |
|  | AU4.1 | 305 | 14393 | 3.6774 | 0.8875 | 460.2174 | 99.4094 |
|  | AU4.2 | 307 | 14376 | 3.6799 | 0.8876 | 430.1034 | 99.4087 |
|  | AU4.3 | 306 | 14379 | 3.6811 | 0.8882 | 472.4348 | 99.3880 |
|  | AU4.4 | 305 | 14407 | 3.6771 | 0.8874 | 429.5000 | 99.4170 |
|  | AU4.5 | 306 | 14412 | 3.6807 | 0.8877 | 436.5357 | 99.4033 |
|  | AU5.1 | 22 | 6059 | 0.2479 | 0.0758 | 55.0000 | 99.8019 |
|  | AU5.2 | 23 | 6032 | 0.2490 | 0.0762 | 62.0000 | 99.7845 |
|  | AU5.3 | 21 | 6038 | 0.2395 | 0.0739 | 87.0000 | 99.8013 |
|  | AU5.4 | 22 | 6008 | 0.2442 | 0.0752 | 55.0000 | 99.8003 |
|  | AU5.5 | 22 | 6079 | 0.2286 | 0.0696 | 44.0000 | 99.8026 |
| Winter | WI1.1 | 956 | 63596 | 5.1495 | 0.9803 | 1122.7941 | 99.8318 |
|  | WI1.2 | 956 | 63600 | 5.1485 | 0.9803 | 1121.4545 | 99.8349 |
|  | WI1.3 | 956 | 63624 | 5.1512 | 0.9803 | 1126.6250 | 99.8350 |
|  | WI1.4 | 955 | 63623 | 5.1498 | 0.9803 | 1112.5294 | 99.8365 |
|  | WI1.5 | 955 | 63595 | 5.1508 | 0.9803 | 1125.6250 | 99.8349 |
|  | WI2.1 | 542 | 16934 | 3.3821 | 0.8967 | 666.9592 | 99.0729 |
|  | WI2.2 | 539 | 16934 | 3.3760 | 0.8963 | 665.9681 | 99.0847 |
|  | WI2.3 | 541 | 16950 | 3.3800 | 0.8966 | 672.6774 | 99.0737 |
|  | WI2.4 | 542 | 16943 | 3.3821 | 0.8966 | 667.9375 | 99.0793 |
|  | WI2.5 | 542 | 16945 | 3.3773 | 0.8962 | 651.2952 | 99.1030 |
|  | WI3.1 | 438 | 9765 | 3.8246 | 0.8967 | 569.4490 | 98.8326 |
|  | WI3.2 | 441 | 9784 | 3.8192 | 0.8956 | 576.7200 | 98.8042 |
|  | WI3.3 | 439 | 9732 | 3.8291 | 0.8968 | 562.8654 | 98.8286 |
|  | WI3.4 | 441 | 9758 | 3.8194 | 0.8958 | 574.4000 | 98.8112 |
|  | WI3.5 | 437 | 9770 | 3.8244 | 0.8962 | 548.0185 | 98.8741 |
|  | WI4.1 | 476 | 4272 | 4.7197 | 0.9725 | 696.1392 | 95.6227 |
|  | WI4.2 | 470 | 4281 | 4.7123 | 0.9727 | 680.7975 | 95.7253 |
|  | WI4.3 | 474 | 4275 | 4.7103 | 0.9722 | 709.0135 | 95.6257 |
|  | WI4.4 | 473 | 4291 | 4.7190 | 0.9726 | 706.8333 | 95.7120 |
|  | WI4.5 | 470 | 4255 | 4.7164 | 0.9726 | 704.5493 | 95.6992 |
|  | WI5.1 | 47 | 848 | 3.0181 | 0.9274 | 62.0000 | 98.2311 |
|  | WI5.2 | 46 | 850 | 3.0105 | 0.9274 | 57.3750 | 98.3529 |
|  | WI5.3 | 46 | 838 | 3.0092 | 0.9265 | 53.3333 | 98.5680 |
|  | WI5.4 | 45 | 844 | 3.0182 | 0.9280 | 51.1111 | 98.6967 |
|  | WI5.5 | 47 | 834 | 3.0141 | 0.9269 | 57.1111 | 98.3213 |
